# Loss of miR-424 and miR-503 promotes decidualization of human endometrial stromal cells by increasing SCARA5 expression

**DOI:** 10.1007/s00795-025-00431-5

**Published:** 2025-03-14

**Authors:** Tetsu Yamaguchi, Masashi Takamura, Hideno Tochigi, Yumi Mizuno, Yosuke Mizuno, Tomomi Sato, Shunsuke Tamaru, Kazuya Kusama, Kazuhiro Tamura, Yoshimasa Kamei, Takeshi Kajihara

**Affiliations:** 1 Department of Obstetrics and Gynecology, Saitama Medical University, 38 Morohongo, Moroyama, Iruma-gun, Saitama, Japan; 2 Division of Biomedical Research Center, Saitama Medical University, 38 Morohongo, Moroyama, Iruma-gun, Saitama, Japan; 3 Department of Anatomy, Saitama Medical University, 38 Morohongo, Moroyama, Iruma-gun, Saitama, Japan; 4 Department of Endocrine Pharmacology, Tokyo University of Pharmacy and Life Sciences, 1432-1 Horinouchi, Hachioji, Tokyo Japan

**Keywords:** Endometrial decidualization, Forkhead box protein O1, miR-424, miR-503, Scavenger receptor class A member 5

## Abstract

This study aims to investigate the function of miR-424 and miR-503, identified as putative regulatory miRNAs of *FOXO1*, a key factor for decidualization. The expression of both miR-424 and miR-503 in human endometrial stromal cells (HESCs) were measured before and after decidualization. Then, HESCs were transfected with both miR-424 and miR-503 before decidualization. Quantitative reverse transcription PCR, actin staining analysis, migration assay, fluorescence immunostaining, and luciferase assay were performed. MiR-424 and miR-503 expression was decreased after decidualization. Overexpression of both miR-424 and miR-503 inhibited major decidual maker genes, including *FOXO1*, *PRL*, *IGFBP1*, *WNT4,* and *SCARA5,* and altered F-actin’s subcellular distribution from the periphery to all over the cytoplasm, concomitantly increasing cell mobility. Moreover, immunohistochemical analysis revealed overexpression of both miRNAs resulted in FOXO1 protein accumulation in the cytoplasm. Knocking down FOXO1 decreased SCARA5 expression, revealing SCARA5 is a downstream target of FOXO1. In addition, a luciferase reporter assay confirmed that the 3′-untranslated region of *FOXO1* mRNA is targeted by miR-424. These results suggest that both miRNAs may play an important role in endometrial decidualization by regulating transcriptional activity of FOXO1, which alters decidualization-related gene expression such as SCARA5.Abstract: Journal standard instruction requires an unstructured abstract; hence structured abstract changed to unstructured.Thank you for the correction. I approve this change.

**Supplementary Information:**

The online version contains supplementary material available at 10.1007/s00795-025-00431-5.

## Introduction

Decidualization is a process which consists on the dramatic morphological and functional differentiation from uterine human endometrial stromal cells (HESCs) into decidual cells. During this process, HESCs are transformed from elongated fibroblast-like mesenchymal cells into round, epithelioid-like cells [1]. This dynamic change is driven by progesterone and locally produced cyclic adenosine monophosphate (cAMP) [2, 3]. Successful decidualization is essential for implantation and normal pregnancy; thus, impairments in this process can lead to implantation failure, miscarriage, and other obstetrical complications [1].

When HESCs differentiate into decidualized cells, they show a molecular signature of mesenchymal–epithelial transition characterized by changes in the expression of various genes, including the induction of *HOXA10*, *HOXA11*, *FOXO1*, *WNT4*, *IGFBP1*, and *PRL* [4]. Among them, we previously focused on FOXO1 as a key factor for decidualization. FOXO1 has been shown to regulate differentiation, menstrual shedding, and feto-maternal protection against oxidative damage during pregnancy [5]; however, the regulation of FOXO1 expression and the mechanism underlying the morphological changes of HESCs remain unclear. Takano et al. identified the genes induced in decidualized HESCs in a FOXO1-dependent manner [6]. In the present study, we focused on scavenger receptor class A member 5 (SCARA5), the 2nd highest inducible gene in that study. SCARA5 is a newly discovered scavenger receptor shown to act as a tumor suppressor gene in several types of cancer [7, 8]. In addition, some studies have shown the inhibitory role of SCARA5 in the epithelial–mesenchymal transition [9, 10], indicating that this gene can be a key factor in the dynamic morphological change occurring during decidualization.

MicroRNAs (miRNA), small non-coding RNAs approximately 21–25 nucleotides which regulate gene expression by silencing mRNA translation or decreasing its stability [11], have been involved in several biological processes, including cell differentiation and proliferation as well as organ development and metabolism [12]. Previously, we identified five miRNAs whose expression was altered upon decidualization (< 0.5-fold) [11]. Among them, two miRNAs (miR-424 and miR-503) putatively target *FOXO1* expression [11]. However, the role of miR-424 and miR-503 in decidualization has not been well investigated.

Thus, this study aimed to investigate the role of the two miRNAs in *FOXO1* expression and its effects on *SCARA5* expression during HESC decidualization.

## Materials and methods

### Tissue samples and culture

The collection and use of tissue samples in this study were conducted in accordance with the protocol approved by the Institutional Review Board of Saitama Medical University Hospital (research approval numbers: 18085, 2024007). All specimens were obtained with written informed consent from the subjects. HESCs were obtained from patients who had undergone hysterectomy for benign tumors such as uterine myoma. All patients had regular menses and had not received hormone therapy for 3 months prior to surgery. HESCs were sterilely collected after hysterectomy, isolated, and cultured according to a previously reported protocol [13–15]. Briefly, endometrial specimens were collected in Dulbecco's Modified Eagle Medium /Nutrient Mixture F-12 (D-MEM/F-12 Themo Fisher Scientific, Waltham, MA, USA) containing 10% fetal bovine serum (FBS Nichirei Biosciences, Tokyo, Japan) and 1% antibiotic–antimycotic (Maintenance Medium; Thermo Fisher Scientific, Waltham, MA, USA). After enzymatic digestion with DNase I (Sigma-Aldrich, St. Louis, MO, USA) and collagenase Ia (Sigma-Aldrich), stromal cells were separated from epithelial cells and cultured in maintenance medium for decidualization stimuli. After reaching confluency, to stimulate decidualization, the medium was changed to D-MEM/F-12 medium containing 2% FBS mixed with 0.5 mM 8-bromo-cyclic adenosine monophosphate (8-br-cAMP, Sigma-Aldrich) and 10^−6^ M medroxyprogesterone acetate (MPA, Sigma-Aldrich). Decidualization stimulation was continued for either 3, 6, or 7 days depending on the experiment. When lasting for 6–7 days, the medium was changed 3 days after the beginning of decidualization stimulation.

### Evaluation of decidualization

Decidualization was evaluated by both morphological changes and the mRNA expression levels of *PRL* and *IGFBP1*. Images were captured using an ECLIPSE TS100 phase-contrast microscope (NIKON, Tokyo, Japan) with a 100 × objective lens (numerical aperture of the objective: 0.75).

### Total RNA extraction and quantitative reverse transcription PCR

For extracting total RNA from HESCs, an miRNeasy Mini kit (Qiagen, Hilden, Germany) was used. The total RNA concentration was quantified by NanoDrop (Thermo Fisher Scientific). Total RNA was reversely transcribed with PrimeScript™ RT reagent kit (Takara Bio, Shiga, Japan).

The PowerUP™ SYBR™ Green Master Mix (Thermo Fisher Scientific) was used for qRT-PCR. The primers used for each gene are shown in Table 1. TaqMan MicroRNA assays (Thermo Fisher Scientific) for miR-424 and miR-503 were used for reverse transcription and amplification. Each qRT-PCR was performed using a PikoReal™ 96 real-time PCR system (Thermo Fisher Scientific). The mRNA expression levels of *PRL*, *IGFBP1*, *WNT4*, *FOXO1*, and *SCARA5* were calculated relative to *GAPDH* and the expression levels of miR-424 and miR-503 were calculated relative to *U6* (Thermo Fisher Scientific) by the 2^−ΔΔct^ method.

**Table 1:** Primer sequences for qRT-PCR

Primer name	Primer sequence (5′–3′)
FOXO1-forward	ATTCGGAATGACCTCATGGA
FOXO1-reverse	TTTTAAGTGTAACCTGCTCACTAACC
PRL-forward	CTACATCCATAACCTCTCCTCA
PRL-reverse	GGGCTTGCTCCTTGTCTTC
IGFBP1-forward	CTGCGTGCAGGAGTCTGA
IGFBP1-reverse	CCCAAAGGATGGAATGATCC
WNT4-forward	CATGCAACAAGACGTCCAAG
WNT4-reverse	AAGCAGCACCAGTGGAATTT
SCARA5-forward	CATGCGTGGGTTCAAGGTG
SCARA5-reverse	CCATTCACCAGGCGGATCAT
GAPDH-forward	CGACCACTTTGTCAAGCTCA
GAPDH-reverse	AGGGGTCTACATGGCAACTG

### miRNA transfection

HESCs were cultured in 6-well plates. According to the manufacturer's protocol, Lipofectamine^®^ 2000 (Thermo Fisher Scientific) was mixed with hsa-miR-503 mimic (Thermo Fisher Scientific) and hsa-miR-424 mimic (Thermo Fisher Scientific), and added to each well. Simultaneously, control miRNA mimic (nc-mimic; Thermo Fisher Scientific) was used as a negative control. At 6 h or overnight after transfection, the medium was changed to D-MEM/F-12 medium containing 2% FBS mixed with 0.5 mM 8-br-cAMP and 10^−6^ M MPA. After 3 days, the medium was changed. The morphological evaluation and RNA extraction were performed on day 6.

### siRNA transfection

HESCs were cultured in 6-well plates. The Xfect™ RNA Transfection Reagent (Takara Bio) was mixed with siRNA for *SCARA5* (siR-SCARA5, SASI_Hs01_00076732, Thermo Fisher Scientific) or siRNA for *FOXO1* (siR-FOXO1, SASI_Hs01_00108488, Thermo Fisher Scientific) and added to each well according to the manufacture’s protocol. As negative control, non-targeting siRNA (Thermo Fisher Scientific) was used instead of siR-SCARA5 or siR-FOXO1. At 4 h or overnight after transfection, the medium was changed to D-MEM/F-12 medium containing 2% FBS mixed with 0.5 mM 8-br-cAMP and 10^−6^ M MPA. RNA extraction was performed on day 3.

### Luciferase assay

This experiment was approved by the Secretariat of the Safety Committee for Recombinant DNA Experiments (research approval numbers: 1557) and the Cartagena Protocol on Biosafety to the Convention on Biological Diversity. We used the pmirGLO Dual Luciferase miRNA Target Expression Vector (Promega, Madison, WI, USA). The vector was linearized using PrimeSTAR GXL Premix Fast, Dye plus (Takara Bio), the linearization primer-F (5′-GTCTAGAGTCGACCTGCAGGC-3′), and primer-R (5′-TCGAGGCTAGCGAGCTCGT-3′) by PCR. The miR-424 candidate targeting sequence of FOXO1 gene (5′-TGTGTGCAGGTTATGTGCTGCTG-3′) or its randomly aligned control sequence (5′-TTTCTTGGGGGTCGTATGGCAGTG-3′) were inserted into the multi cloning site of the linearized vector and transformed using In-Fusion^®^ Snap Assembly Master Mix with Competent Cells (Takara Bio) at 50 °C for 15 min. The multicloning site is located downstream of the firefly luciferase gene. Competent cells and vectors were mixed and incubated on Lysogeny Broth (LB) medium plates containing ampicillin overnight.

We collected colonies from each plate and incubated them in LB medium at 37 °C with shaking overnight. Next day, the vector was purified by PureYield™ Plasmid Midiprep System (Promega). The resulting vectors were named FOXO1-miR424-target-wt and FOXO1-miR424-target-mut, respectively.

For transfection, 500 ng of vector and 1 nM of hsa-miR-424 mimic and hsa-miR-503 mimic per well were added to 24-well plates with immortalized HESCs [16], and Lipofectamine^®^ 3000 (Thermo Fisher Scientific) was used according to the manufacturer’s protocol. As a control for the transfection of hsa-miR-424 and hsa-miR-503 mimics, nc-mimic were transfected instead. After 48 h, the cells were harvested using the Passive Lysis Buffer in Dual Luciferase Reporter Assay System (Promega) and aliquoted onto 96-well plates; then, firefly and Renilla luciferase activities were sequentially measured using VariioSkanFlash (Thermo Fisher Scientific). The relative firefly luciferase activity was calculated by normalizing to the Renilla luciferase activity.

### Fluorescence immunostaining for FOXO1

HESCs were grown on coverslips and co-transfected with miR-424 and miR-503 mimics, or nc-mimic. At 4 h or overnight after transfection, a medium change was performed for decidualization stimulation. After 6 days, the cover glass was washed with phosphate-buffered saline (PBS, Nacalai tesque, Kyoto, Japan) three times and fixed in 4% paraformaldehyde for 15 min. After washing with PBS, cell membranes were disrupted with 0.5% Triton X-100 (Nacalai tesque) for 10 min and washed with PBS. Cells were blocked with 2% bovine serum albumin (Sigma-Aldrich) in PBS for 1 h, washed with PBS, and incubated with an anti-FOXO1 antibody (1:100 dilution; Cell Signaling Technology, Danvers, MA, USA) overnight. Next day, after washing with PBS, anti-rabbit immunoglobulin G conjugated with Alexa Fluor 488 (1:10,000 dilution; Invitrogen, Waltham, MA, USA) in PBS was incubated as secondary antibody for 1 h at room temperature. After washing with PBS, the mounting medium for fluorescence with DAPI (Vector Laboratories, Newark, CA, USA) was used for nuclear staining. An AxioImager Z2, LSM 710 (Carl Zeiss, Oberkochen, Germany) was used for observation.

### Fluorescence immunostaining for SCARA5

HESCs were grown on coverslips and medium change was performed for decidualization stimulation.

After 6 days, the cover glass was washed with phosphate-buffered saline (PBS, Nacalai tesque, Kyoto, Japan) three times and fixed in 4% paraformaldehyde for 15 min. After washing with PBS, cell membranes were disrupted with 0.5% Triton X-100 (Nacalai tesque) for 10 min and washed with PBS. Cells were blocked with 2% bovine serum albumin (Sigma-Aldrich) in PBS for 1 h, washed with PBS, and incubated with an anti-SCARA5 antibody (1:100 dilution; Thermo Fisher Scientific) for 3 h. After washing with PBS, anti-rabbit immunoglobulin G conjugated with Alexa Fluor 488 (1:5,000 dilution; Invitrogen) in PBS was incubated as secondary antibody for 1 h at room temperature. After washing with PBS, the mounting medium for fluorescence with DAPI (Vector Laboratories) was used for nuclear staining. An AxioImager Z2, LSM 710 (Carl Zeiss) was used for observation.

### Migration assay

HESCs were spread on a 6-well-plate and incubated until cell abundance reached 80% confluency. Then, we co-transfected HESCs with hsa-miR-424 and hsa-miR-503 mimics or nc-mimic with Lipofectamine^®^ 2000. At 4 h or overnight after transfection, a 1000 µL sterile pipette tip was used to scratch the culture plate; then, the medium was changed for decidualization stimulation. Immediately and at 36 h after the scratch, images of the same area were captured using an ECLIPSE TS100 phase-contrast microscope with a 40 × objective lens. The migration distance was quantified by measuring the area without cell attachment using Image J (version 1.53e, https://imagej.nih.gov/ij/) and calculating the changes to the area.

### Actin staining analysis

HESCs were grown overnight on coverslips and co-transfected with hsa-miR-424 and hsa-miR-503 mimics, or nc-mimic. Then, the medium was changed for decidualization stimulation for 7 days. After washing with PBS three times, the cover glass was fixed with 4% paraformaldehyde for 15 min. After washing with PBS, the cells were stained with Alexa Fluor™ 555 Phalloidin (Thermo Fisher Scientific) for 30 min at room temperature. Next, cells were washed with PBS and DAPI staining was performed for nuclear staining. An AxioImager Z2, LSM 710 was used for observation. The lengths of the major and minor axes of the actin sequence were measured using ImageJ and the ratio of actin filament’s width to its length were calculated.

### Statistical analysis

Statistical analysis was performed by Welch’s *t* test or Tukey test in Excel (version 2020, Microsoft, Redmond, WA, USA). Data are indicated as mean ± standard error. *P* < 0.05 was considered to indicate a significant difference.

## Results

### Morphological and biochemical changes upon decidualization

The efficiency of HESC decidualization was confirmed by morphological and biochemical markers. As reported previously [3], the cell shape was fibroblast-like on light microscopy when cultured without treatment (Supplemental Fig. 1a). However, under treatment with 8-br-cAMP and MPA, the shape of HESCs became larger and rounder (Supplemental Fig. 1b). The mRNA expression of *WNT4*, *IGFBP1* and *PRL* in treated HESCs was significantly higher than in non-treated control cells (Supplemental Fig. 1c–e).

### Downregulation of miR-424 and miR-503 expression upon decidualization

The differential gene expression of these two miRNAs was confirmed by qRT-PCR analysis. As shown in Supplemental Fig. 2, a significant decrease in the expression of both miR-424 and miR-503 was observed in HESCs with decidualization in comparison to control cells.

### miR-424 and miR-503 overexpression decreases the expression of decidual markers

To explore the potential role of both miR-424 and miR-503 in HESC decidualization, we co-transfected HESCs with miR-424 and miR-503 mimics or nc-mimic prior to decidualization for 6 days.

**Fig. 1:**
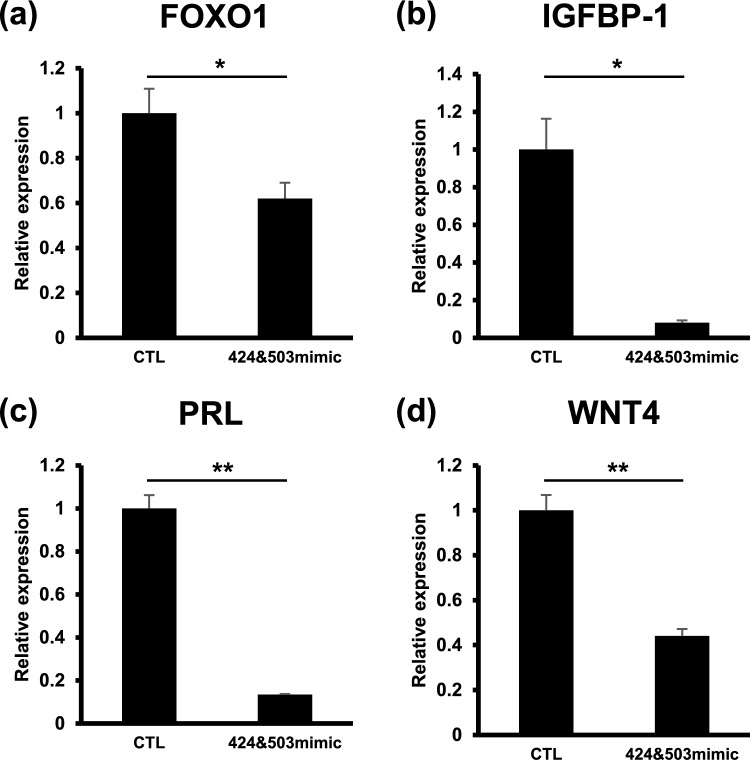
Downregulation of mRNA expression of decidualization-associated genes by miR-424 and miR-503 overexpression during decidualization. qRT-PCR analysis of *FOXO1* (**a**), *IGFBP-1* (**b**), *PRL* (**c**) and *WNT4* (**d**) transcript levels in HESCs transfected with both hsa-miR-424 and hsa-miR-503 mimics (424 and 503mimic) or nc-mimic (CTL), followed by stimulation with 8-br-cAMP and MPA for 6 days. Data indicate mean ± standard error. *P < 0.05, **P < 0.01

*FOXO1* expression was measured by qRT-PCR analysis. Compared to decidualized HESCs transfected with nc-mimic, *FOXO1* expression in decidualized HESCs was significantly inhibited by the addition of the hsa-miR-424 and hsa-miR-503 mimics. Similarly, the expression of *IGFBP1*, *PRL*, and *WNT4* was also markedly repressed by the hsa-miR-424 and hsa-miR-503 mimics (Fig. 1).

### FOXO1 silencing suppresses SCARA5 expression

**Fig. 2:**
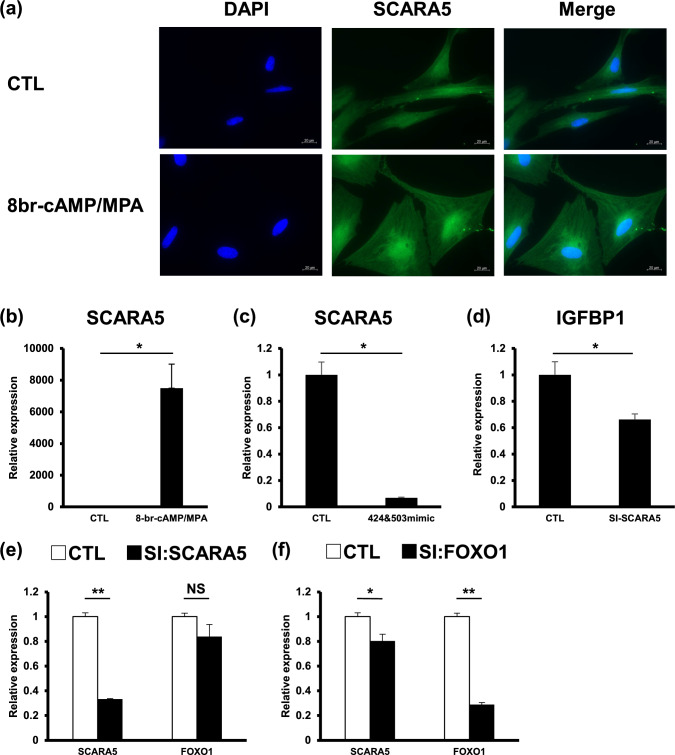
SCARA5 is a downstream target of FOXO1. SCARA5 localization in decidualized HESCs (**a**). qRT-PCR analysis of *SCARA5* transcript levels in HESCs after decidualization (**b**) or transfected with both hsa-miR-424 and hsa-miR-503 mimics (424 and 503 mimic) or nc-mimic (CTL) (**c**), followed by stimulation with 8-br-cAMP and MPA for six days. qRT-PCR analysis of *IGFBP1* transcript levels in HESCs transfected with siR-SCARA5 (**d**). HESCs were transfected with siR-FOXO1 (**e**) or siR-SCARA5 (**f**) and decidualized by treatment with 8-br-cAMP and MPA for 3 days. The relative expression levels of either *FOXO1* or *SCARA5* mRNA (SI) against control (CTL) are indicated. qRT-PCR analysis for the *FOXO1* knockdown (**e**). qRT-PCR analysis for the *SCARA5* knockdown (**f**). Data indicate mean ± standard error. *P < 0.05, **P < 0.01, *NS* not significant. Scale bar, 20 µm

Immunocytochemistry revealed the expression of SCARA5 was markedly increased around the nuclei of decidualized HESCs compared to non-decidualized HESCs (Fig. 2a). This was also confirmed at mRNA level (Fig. 2b) and addition of the hsa-miR-424 and hsa-miR-503 mimics significantly decreased *SCARA5* mRNA expression (Fig. 2c). Together with the result of Fig. 1a, this observation suggests that miR-424 and miR-503 regulated the expression of both *FOXO1* and *SCARA5* genes.

The effect of SCARA5 upon decidualization has not been clarified yet, we explored the effect of SCARA5 on decidualization markers such as IGFBP1 and PRL. The expression of *IGFBP1* was slightly reduced in decidualized HESCs stimulated with SCARA5 siRNA (Fig. 2d), whereas *PRL* was not (data not shown).

Next, we speculated that *FOXO1* regulates *SCARA5* mRNA expression in HESCs during decidualization. To verify this hypothesis, we transfected HESCs with siRNAs targeting FOXO1, SCARA5, or non-targeting siRNA. After transfection, HESC cultures were decidualized for 3 days. Knocking down *FOXO1* significantly reduced *SCARA5* mRNA expression whereas knocking down SCARA5 did not alter FOXO1 mRNA expression (Fig. 2e, f). These results indicate that FOXO1 acts as an upstream regulator of SCARA5.

### FOXO1 is a Bona fide target of miR-424

**Fig. 3:**
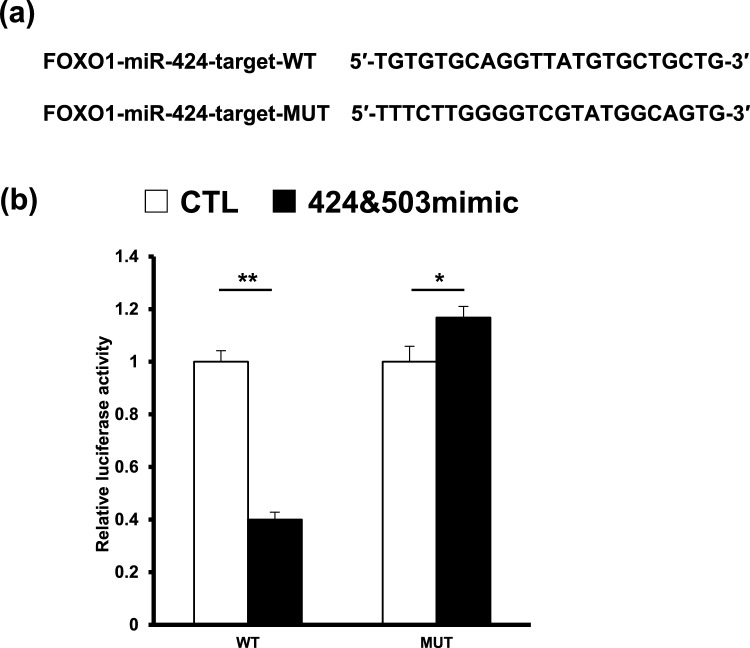
FOXO1 is a direct target of miR-424. **a** Predicted target (top) and mutant (bottom) sequences of miR-424 in the 3′ untranslated region of FOXO1 used in the luciferase assay. **b** Relative luciferase activity using FOXO1-miR-424-target-WT (WT) and FOXO1-miR-424-target-mut (MUT) reporter vectors in decidualized HESCs co-transfected with both hsa-miR-424 and hsa-miR-503 mimics (424 and 503 mimic) or nc-mimic (CTL). Data indicate mean ± standard error. *P < 0.05. **P < 0.01

Further, co-transfection of HESCs with FOXO1-miR424-taget-wt and hsa-miR-424 and hsa-miR-503 mimics decreased luciferase activity compared to co-transfection with nc-mimic. In contrast, cells were transfected with a FOXO1-miR424-target-mut reporter vector did not decrease. This indicated that hsa-miR-424 and hsa-miR-503 mimics both inhibit FOXO1 expression by regulating its 3′ UTR target sequence (Fig. 3).

### miR-424 and miR-503 overexpression inhibits FOXO1’s nuclear localization

**Fig. 4:**
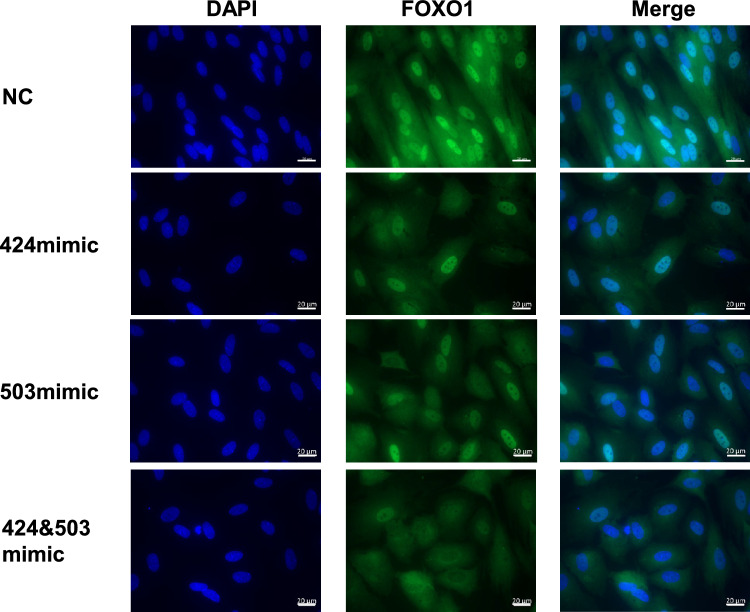
miR-424 and miR-503 overexpression alters FOXO1 localization in decidualized HESCs. The localization of FOXO1 is changed from intranuclear to extranuclear by miR-424 and miR-503 mimic. This effect becomes even stronger when two miRs are added. Scale bar, 20 µm

In this present study, despite the 0.6-fold decrease in *FOXO1* mRNA expression in HESCs with hsa-miR-424 and hsa-miR-503 mimics compared to the control, *SCARA5* mRNA expression was remarkably decreased (Fig. 1a). This could not be entirely explained by the decreased *FOXO1* mRNA expression. Another possible explanation could be changes in the subcellular distribution of FOXO1 protein. Therefore, we examined the expression and localization of FOXO1 protein with immunofluorescence. Interestingly, FOXO1 protein localization in decidualized HESCs changed dramatically from nuclear to cytoplasmatic after miR-424 and miR-503 overexpression (Fig. 4). This translocation was observed even stronger after both miRs were overexpressed compared to HESCs with each miR overexpressed.

### miR-424 and miR-503 enhance migration of decidualized HESCs

**Fig. 5:**
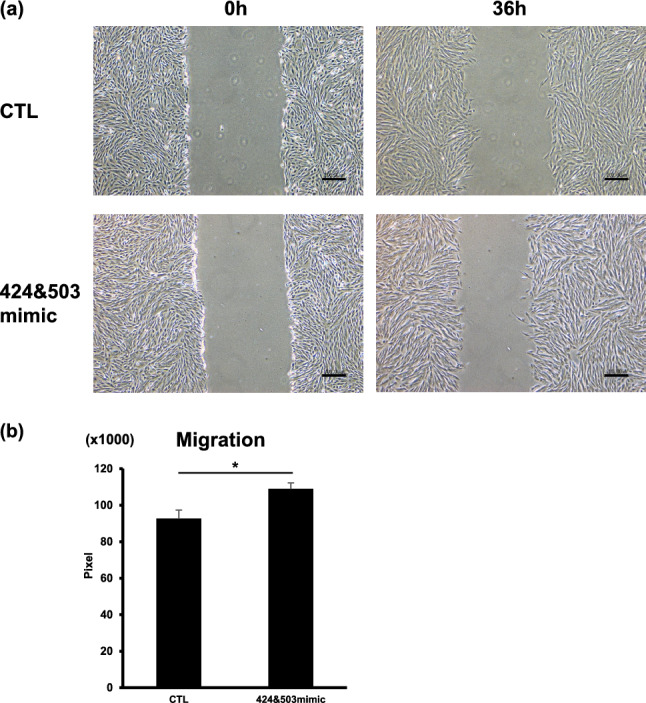
MiR-424 and miR-503 promote HESC motility. Scratch assay using decidualized HESCs co-transfected with both hsa-miR-424 and hsa-miR-503 mimics (424 and 503 mimic) or nc-mimic (CTL) and treated with 8-br-cAMP and MPA for 6 days. **a** Representative images immediately after scratch (0 h) and 36 h later (36 h). **b** Quantification of scratch assay. Scale bar, 200 µm. *P < 0.05

To analyze the effect of both miRNAs on migration, a scratch assay was performed on HESCs transfected with hsa-miR-424 and hsa-miR-503 mimics and treated with decidualizing stimuli for 6 days. Cell motility significantly increased with overexpression of both miRNAs in decidualized HESCs in comparison with the negative control (Fig. 5).

### miR-424 and miR-503 overexpression inhibit the morphological changes occurring in HESCs with decidualization

**Fig. 6:**
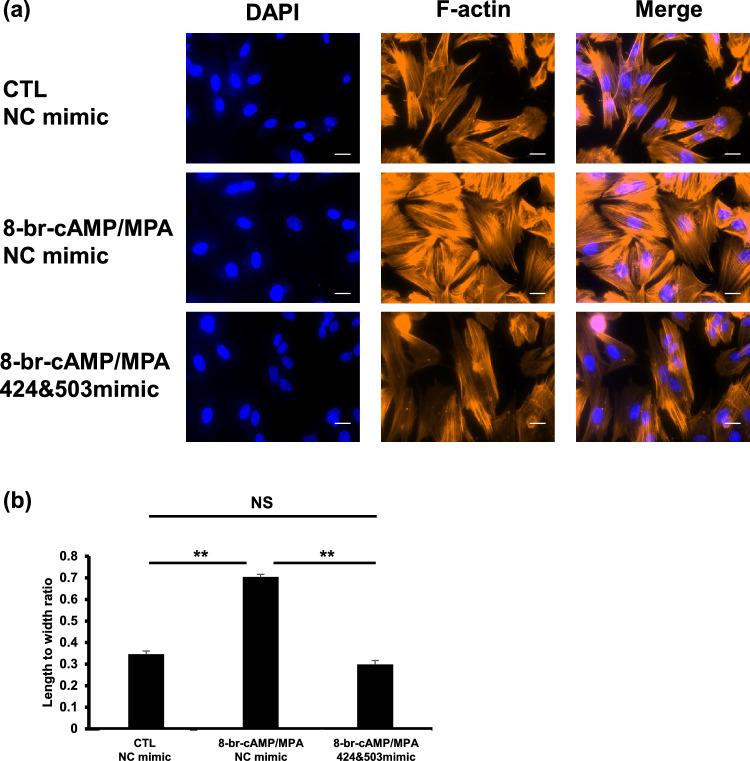
miR-424 and miR-503 inhibit changes in F-actin distribution after decidualization. **a** F-actin is distributed in a spindle-shape in HESCs without decidualization treatment (CTL—NC mimic), whereas it is circumferentially aligned in HESCs with decidualization treatment (8-br-cAMP and MPA for 6 days) transfected with nc-mimic, the negative control (8-br-cAMP/MPA—NC mimic). This change is cancelled in HESCs transfected with hsa-miR-424 and hsa-miR-503 mimics (8-br-cAMP/MPA—424 and 503 mimic). **b** Length to width ratio of actin filament in each HESCs. The ratio of 8-br-cAMP/MPA—424 and 503 mimic is significantly smaller than that of 8-br-cAMP/MPA—NC mimic and close to that of CTL—NC mimic. Scale bar, 20 µm. **P < 0.01

As shown in Fig. 6a, cells transfected with hsa-miR-424 and hsa-miR-503 mimics exhibited a spindle-shaped F-actin disposition and fibroblast-like cell shape whereas the F-actin was circumferentially aligned in cells transfected with the nc-mimic. As shown in Fig. 6b, the length-to-width ratio of the actin sequence was measured using the major axis as the denominator and the minor axis as the numerator, and it was confirmed that HESCs with hsa-miR-424 and hsa-miR-503 mimics had a smaller ratio than nc-mimic. Thus, miR-424 and miR-503 inhibited morphological differentiation of HESCs under decidualization stimuli.

## Discussion

**Fig. 7:**
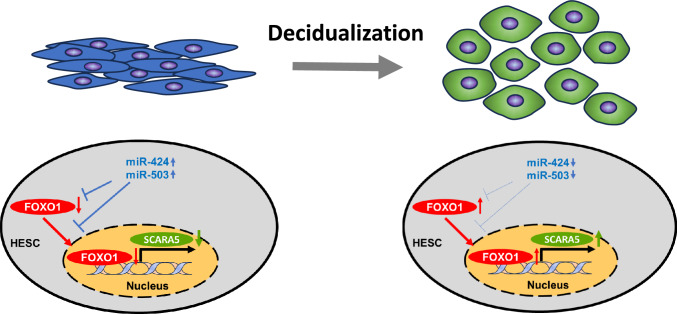
Schematic summary of the changes after decidualization demonstrated in this study. Left, before decidualization. High miR-424 and miR-503 expression levels suppress *FOXO1* expression and inhibit decidualization. Right, after stimulation of decidualization. Decreased miR-424 and miR-503 expression increases FOXO1 protein expression, especially in the nucleus, thus increasing its transcriptional activity, upregulating *SCARA5* expression

In the current study, we confirmed overexpression of miR-424 and miR-503 decreased expression of decidual marker genes, inhibited the morphological transformation, recovered the motility, and induced a shift in FOXO1 localization from the nucleus to the cytoplasm of decidualized HESCs. Knockdown of *FOXO1* or *SCARA5* revealed that *SCARA5* is a downstream target of *FOXO1*. Finally, we confirmed that *FOXO1* is a direct target of miR-424 by luciferase assay. The underlying mechanism of decidualization unveiled in the current study is illustrated in Fig. 7.

Kindly check and confirm Fig. 9 cited in text changed to Fig. 7.Thank you for the correction. Of course, I agree with this change

*FOXO1* is a key factor for decidualization. Although FOXO1 is reportedly regulated by miR-375 during decidualization, it remains unclear whether this regulation is accompanied by direct targeting [18]. Our study demonstrated for the first time the direct regulation of FOXO1 by miRNAs in decidualized HESCs.

Moreover, this study revealed that miR-424 and miR-503 negatively regulate FOXO1 expression in decidualization, which, to our knowledge, had not yet been reported. miR-424 and miR-503 have been indicated to work synergistically. Li et al. reported that the miR-424 and miR-503 cluster is targeted to the 3′ UTR region of Smad7 and Smurf2 mRNAs in breast cancer cells [19] and in colon cancer, it would upregulate Rictor [20]. However, such synergistic function of miRNAs is not always the case in different cells [21]. Thus, these miRNAs appear to have cell-type specific functions [21–23] and our findings might show a unique role of miR-424 and miR-503 in FOXO1 regulation during decidualization.

Several factors can alter the localization of FOXO1. Such factors include beta-casomorphin-7 [24], cyclin-dependent kinase 5 [25], insulin-like growth factor 1 [26], STAT3 [27], and oleic acid [28], but not miRNAs. How miR-424 and miR-503 alter FOXO1 localization remains unclear; thus, future research is warranted to clarify this issue.

We identified a new function of FOXO1 as SCARA5 inducer during decidualization. Previously considered as a tumor suppressor gene [8, 10, 29], only a few studies have reported the involvement of SCARA5 during decidualization. Lucas et al. considered SCARA5 as a selective marker gene that distinguishes decidual cells from progesterone-resistant senescent decidual cells [30]. Duncan et al. found that SCARA5 is the most upregulated gene (181-fold) of all genes expressed in the decidualized endometrium from patients with ectopic pregnancy in comparison with that of the non-decidualized endometrium from other patients with ectopic pregnancy [31]. There have been no published studies on the function of SCARA5 in decidualization. This study revealed knocking down SCARA5 expression suppressed IGFBP1 expression, which suggest SCARA5 may play a role in promoting decidualization. Since several studies have demonstrated that SCARA5 inhibits epithelial–mesenchymal transition in cancer biology [9, 10], SCARA5 may play a role in the morphological changes of HESCs during decidualization. Future research will unveil the specific role of SCARA5 in decidualization.

There are several limitations in the current study. We utilized a well-established in vitro model for decidualization. However, it still lacks epithelial, immune, and vascular endothelial cells, which form the endometrium. This imperfect model can influence the results. The endometrium samples were obtained from patients > 40 years old with gynecological diseases like uterine myoma and endometriosis. This could also affect the experimental results; however, we cannot obtain endometrium from a normal uterus from fertile patients.

## Conclusion

In conclusion, the current study revealed two novel regulators of FOXO1, miR-424 and miR-503, which inhibit the expression of decidualization-related genes and the associated morphological changes. These miRNAs decreased SCARA5 expression mainly through controlling the intracellular localization of FOXO1. Our results indicate that miR-424 and miR-503 play a pivotal role in endometrial decidualization and help understanding of the molecular mechanism of FOXO1 during decidualization.

## Supplementary Information

Below is the link to the electronic supplementary material.

Supplementary file1 Supplemental Fig.1 Changes in cell morphology and altered IGFBP1, PRL, and WNT4 expression in HESCs after decidualization. (a) HESCs have a spindle-shaped morphology before decidualization (CTL). (b) HESCs show a paving stone-like morphology after decidualization with 8-br-cAMP and MPA for six days. qRT-PCR analysis of, IGFBP1 (c), PRL (d), and WNT4 (e). Scale bar, 200 µm. Data indicate mean ± standard error. **P < 0.01 (PPTX 1335 KB)

Supplementary file2 Supplemental Fig.2 Downregulation of miR-424 and miR-503 expression after decidualization. qRT-PCR analysis of miR-424 (a) and miR-503 (b) expression, both are significantly downregulated in HESCs after decidualization treatment with 8-br-cAMP and MPA for six days. Data indicate mean ± standard error. **P < 0.01 (PPTX 43 KB)

## Data Availability

The data sets obtained during the current study are available from the corresponding author on reasonable request.
