# Supplementary material for: Loss of miR-424 and miR-503 promotes decidualization of human endometrial stromal cells by increasing SCARA5 expression
Source: Med Mol Morphol. 2025 Mar 14;58(4):270–80. doi: 10.1007/s00795-025-00431-5 (PMC12644211; doi:10.1007/s00795-025-00431-5)
Supplement: Supplementary file 1 — Supplementary file1 Supplemental Fig.1 Changes in cell morphology and altered IGFBP1, PRL, and WNT4 expression in HESCs after decidualization. (a) HESCs have a spindle-shaped morphology before decidualization (CTL). (b) HESCs show a paving stone-like morphology after decidualization with 8-br-cAMP and MPA for six days. qRT-PCR analysis of, IGFBP1 (c), PRL (d), and WNT4 (e). Scale bar, 200 µm. Data indicate mean ± standard error. **P < 0.01 (PPTX 1335 KB) [file 795_2025_431_MOESM1_ESM.pptx]

## Slide 1
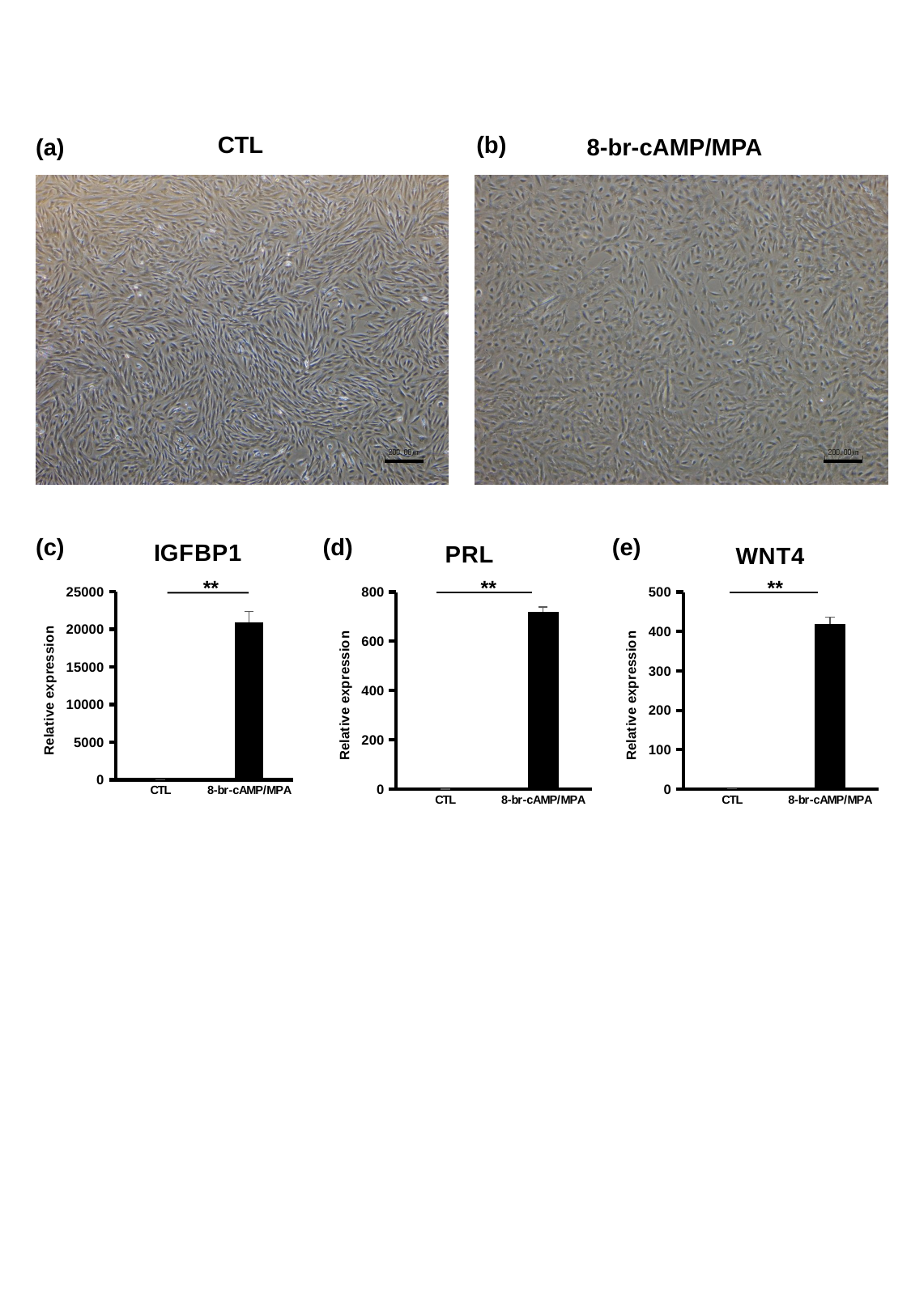

CTL
(b)
(a)
8-br-cAMP/MPA
(e)
**
### Chart: IGFBP1
| Category | |
|---|---|
| CTL | 0.9999999999999999 |
| 8-br-cAMP/MPA | 20952.77997183638 |(c)
### Chart: PRL
| Category | |
|---|---|
| CTL | 1.0 |
| 8-br-cAMP/MPA | 719.0544961025648 |(d)
### Chart: WNT4
| Category | |
|---|---|
| CTL | 1.0 |
| 8-br-cAMP/MPA | 419.46477484701495 |**
**
