# Supplementary figures and images for: Loss of miR-424 and miR-503 promotes decidualization of human endometrial stromal cells by increasing SCARA5 expression
Source: Med Mol Morphol. 2025 Mar 14;58(4):270–80. doi: 10.1007/s00795-025-00431-5 (PMC12644211; doi:10.1007/s00795-025-00431-5)

## Slide 1
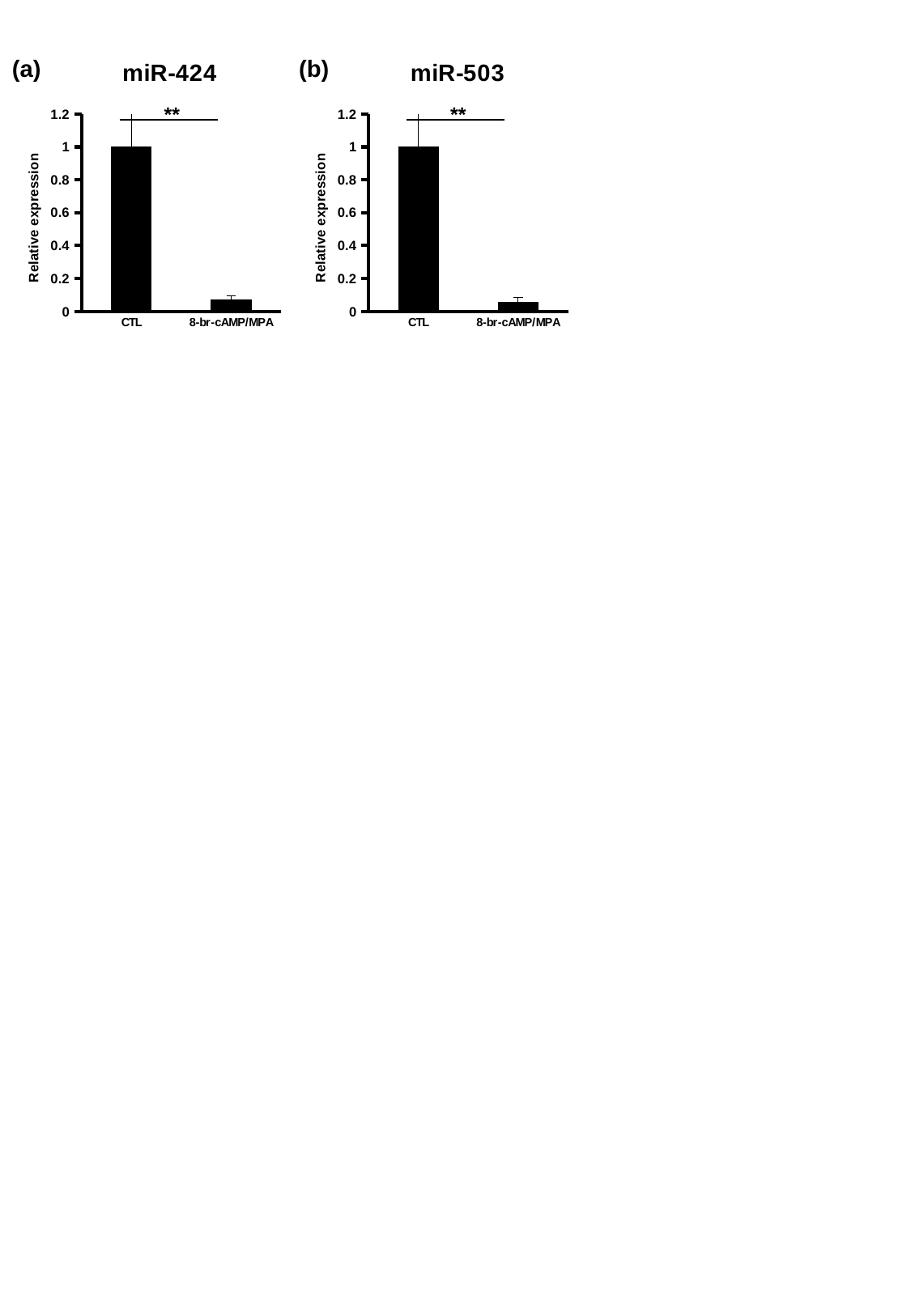

### Chart: miR-424
| Category | |
|---|---|
| CTL | 1.0 |
| 8-br-cAMP/MPA | 0.07043574034174183 |(a)
### Chart: miR-503
| Category | |
|---|---|
| CTL | 1.0 |
| 8-br-cAMP/MPA | 0.054882253115517414 |(b)
**
**

Supplement: Supplementary file 2 — Supplementary file2 Supplemental Fig.2 Downregulation of miR-424 and miR-503 expression after decidualization. qRT-PCR analysis of miR-424 (a) and miR-503 (b) expression, both are significantly downregulated in HESCs after decidualization treatment with 8-br-cAMP and MPA for six days. Data indicate mean ± standard error. **P < 0.01 (PPTX 43 KB) [file 795_2025_431_MOESM2_ESM.pptx]
